# FLASH Radiotherapy in Lung Cancer: Translational Mechanisms, Preclinical Evidence, and Barriers

**DOI:** 10.7759/cureus.101091

**Published:** 2026-01-08

**Authors:** Mariah Abraham, Abdulqadir J Nashwan

**Affiliations:** 1 Nursing &amp; Midwifery Research Department, Hamad Medical Corporation, Doha, QAT

**Keywords:** flash rt, lung cancer, radiotherapy, tumor, uhdr

## Abstract

This narrative review aims to compile the preclinical and translational information about FLASH radiotherapy (FLASH-RT) as a lung cancer therapy. Experimental studies on animal and ex vivo human models have shown that FLASH-RT reduces lung fibrosis and inflammation while maintaining tumor control. The "FLASH effect" is most likely brought on by immunological remodeling, reactive oxygen species (ROS) control, and brief oxygen deprivation. Proton pencil beam scanning (PBS), Bragg peak modulation, and adaptive planning are some of the innovative techniques that have contributed to improvements in high-dose-rate delivery accuracy. When it comes to real-time dosimetry, mobility management, and the standardization of dose-rate data, however, there are still hurdles to encounter. It is necessary to do more research that will include several disciplines, including biology, physics, and clinical innovation, to facilitate safe clinical translation. This review seeks to elucidate existing issues, identify information deficiencies, and inform future research and clinical trial design for FLASH-RT-based lung cancer treatments.

## Introduction and background

**Figure 1:**
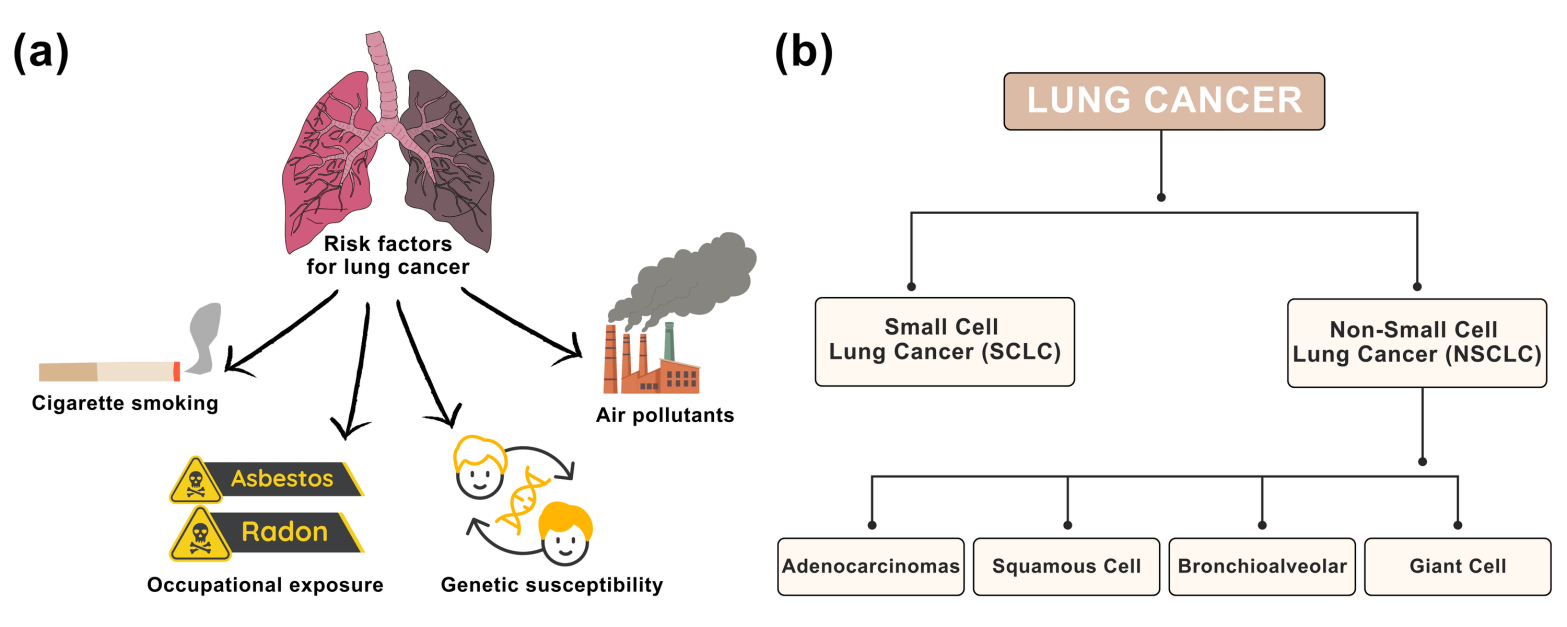
Overview of lung cancer risk factors and classification. (a) Major lung cancer risk factors. (b) Classification of lung cancer into SCLC and NSCLC with major subtypes. Credit: image created by the first author, Mariah Abraham.

Lung cancer is one of the most frequent cancers and still the most significant cause of cancer-related death worldwide. Approximately 2.2 million new patients are diagnosed annually, with a five-year mortality rate exceeding 75%, highlighting the disease's aggressive nature and the limitations of existing therapeutic options [1]. As illustrated in Figure 1, cigarette smoking, exposure to occupational carcinogens like radon and asbestos, air pollution, and genetic susceptibility are among the factors that contribute to lung cancer risk [2,3]. The burden of lung cancer is unevenly distributed across communities, with significant geographic, ethnic, and gender differences in incidence and outcomes [4]. These disparities are due to inequalities in environmental exposures, job hazards, socioeconomic position, healthcare access, and lifestyle habits. Notably, some epidemiological studies indicate that women may be more likely than men to develop lung cancer at equivalent levels of carcinogenic exposure, possibly due to biological, hormonal, or genetic variables influencing susceptibility and disease progression [4].

As illustrated in Figure 1, lung cancer is divided into two primary histological types: small-cell lung cancer (SCLC) and non-SCLC (NSCLC) [5,6]. NSCLC accounts for approximately 80% of all lung cancers and has various histological subtypes, including adenocarcinoma, squamous cell carcinoma, bronchioalveolar carcinoma, and giant cell carcinoma [6]. These subtypes differ significantly in terms of molecular features, growth behavior, and therapeutic response, all of which contribute to disease heterogeneity and complexity. Treatment decisions are primarily stage-dependent. Early-stage NSCLC (stages I-II) is typically treated with surgical resection, frequently with curative intent. Patients with locally advanced or metastatic illness (stages III-IV) often require non-surgical methods, with radiation treatment playing a significant role [7]. Furthermore, lung cancer in the older population has emerged as a growing clinical issue, with over 65% of patients diagnosed after the age of 65 [8].

Radiotherapy (RT) is thus a cornerstone of lung cancer management, with around 60%-70% of patients requiring it at some time during their disease's progression, either as a definitive, adjuvant, or palliative modality. RT exerts its therapeutic effect primarily by inducing DNA damage, leading to tumor cell death or an irreversible growth arrest. However, radiation-induced damage to uninvolved lung tissue sometimes limits the effectiveness of RT [9-11]. This constraint is still one of the most serious obstacles in anticancer RT [12]. Although modern radiation technologies such as tomotherapy, proton RT, and volumetric modulated arc therapy (VMAT) have improved dose conformity and reduced exposure to healthy tissues, their ability to significantly enhance intrinsic tumor control remains limited, emphasizing the need for novel strategies to improve the therapeutic ratio in lung cancer treatment [13].

To address these limitations, FLASH-RT has emerged as a revolutionary treatment [14]. The FLASH concept originated in 1958, when Kirby-Smith and Dolphin observed reduced chromosomal damage under ultra-high-dose-rate (UHDR) electron irradiation [15]. Early work between 1959 and 1982 explored UHDR irradiation using rodent skin models. Researchers showed that dose rates above 2 × 10⁵ Gy/s reduced skin toxicity and confirmed significantly less tail necrosis in rats at pulse dose rates above 10⁵ Gy/s [16]. After this period, research on FLASH remained largely inactive until 2014, when Favaudon et al. demonstrated that UHDR irradiation (60 Gy/s) halted pulmonary fibrosis without compromising tumor control [17]. FLASH-RT is characterized by a single UHDR of 30-100 Gy/s (commonly >40 Gy/s) in less than 500 ms [18-20]. Several preclinical studies have demonstrated that FLASH-RT shows encouraging outcomes by minimizing normal tissue toxicity while preserving tumor control [21]. Pronounced respiratory motion in lung tumors results in dose-delivery uncertainties during conventional RT (CONV-RT). The UHDR and millisecond-scale administration of FLASH-RT significantly diminish motion-induced inaccuracies in thoracic therapies [14,20]. In 2018, Switzerland was the first country to have a human patient undergo FLASH-RT. However, the biological mechanisms underlying its ability to spare normal tissue remain poorly understood [22].

Preclinical studies in mice, minipigs, and ex vivo human lung models have since investigated the biological and dosimetric parameters such as dose rate, pulse structure, modality, and tissue type, following the renewed interest sparked by the groundbreaking 2014 study by Favaudon et al. [23,24]. Among various determinants, tumor hypoxia plays a crucial role in radioresistance. Hypoxia continues to limit treatment effectiveness, even though conventional fractionated RT depends on reoxygenation between doses [25]. This radioresistance arises because limited oxygen diffusion within tumors prevents the stabilization of radiation-induced DNA damage, thereby allowing cancer cells in hypoxic regions to survive and recover after irradiation [26]. It also activates the hypoxia-inducible factor (HIF) pathways, which further help cells survive [27]. Current models lack realistic cellular and oxygen conditions; additional research is required to understand the FLASH impact [28].

Given these insights, one of the most promising clinical contexts for FLASH-RT is lung cancer. Large hypoxic fractions and heterogeneous oxygenation are common characteristics of lung tumors, which significantly increase their resistance to CONV-RT and negatively impact the prognosis of patients [29]. Eighty percent to 85% of cases of lung cancer are NSCLC, which is often detected at a late, incurable stage [30]. Despite advances in proton therapy, immuno-RT, and image-guided RT, normal tissue toxicity and dose-limiting pulmonary complications remain significant barriers [31]. Preclinical models indicate that FLASH-RT mitigates such toxicity, particularly radiation-induced fibrosis and pneumonitis, by employing UHDR [32]. Animal models that demonstrate FLASH-RT's ability to lower pulmonary toxicity without compromising tumor control have raised interest in the therapeutic translation of the drug [33]. The lung is an excellent site for studying the FLASH effect due to its high sensitivity to both acute and long-term radiation damage. From a technology perspective, accurate image guidance is essential to the safe transfer of these findings into medical use. To provide precise dosing and real-time monitoring, El Naqa et al. emphasized the need to combine cone beam computed tomography (CBCT), MRI-linear accelerator (linac), PET, Cherenkov, and acoustic imaging. When combined with artificial intelligence (AI) powered visual guiding, these modalities could significantly ease targeting and mapping tissue oxygenation [34].

The incorporation of FLASH-RT into lung cancer treatment approaches offers both opportunities and challenges. Key areas of investigation include determining optimal fractionation schemes and dosage rates, examining how hypoxia and the tumor microenvironment interact, and evaluating how these therapies could be integrated with systemic treatments such as chemotherapy and immunotherapy [25]. Furthermore, to accurately deliver FLASH beams in clinical settings, particularly for mobile thoracic targets where respiratory motion adds significant complexity, constant technological advancement is required [35]. Current experimental models do not fully replicate the cellular and oxygen conditions of human lung tissue, limiting the biological interpretation of existing data. Collectively, current evidence suggests that FLASH-RT may surmount enduring radiobiological challenges in lung cancer therapy, possibly improving the therapeutic ratio [36]. There is still a significant shortage of studies exploring fractionated and hyper-fractionated FLASH regimens in tumors, as well as research on volume effects, tumor heterogeneity, treatment combinations, treatment planning, and local control outcomes in lung cancer. This review summarizes the biological basis, preclinical findings, technological developments, and clinical challenges from the existing data on FLASH-RT in the treatment of lung cancer.

## Review

Materials and methods

Search Strategies

From July 16 to 25, 2025, an extensive search was conducted on the two databases: Google Scholar and PubMed. For Google Scholar, the search used combinations of the following terms: FLASH RT in lung cancer, proton, electron, photon, "FLASH RT", aiming to capture studies across different beam modalities. For PubMed, the medical subject headings (MeSH) and Title/Abstract search strategy was (“FLASH radiotherapy”[tiab] OR “ultra-high dose-rate radiotherapy”[tiab] OR “ultrahigh dose rate”[tiab] OR “FLASH RT”[tiab] OR “high dose-rate radiation”[tiab]) AND (“Lung Neoplasms”[MeSH] OR “lungcancer”[tiab] OR “lung carcinoma”[tiab] OR “pulmonary neoplasms”[tiab] OR fields were applied. The Boolean s“NSCLC”[tiab] OR “SCLC”[tiab]).

Eligibility Criteria

The inclusion criteria include peer-reviewed articles, research focusing primarily on FLASH-RT in lung cancer, and articles in English. The exclusion criteria include non-peer-reviewed articles, studies not involving FLASH-RT or not specific to lung cancer, and articles not available in English.

Data Filtration Process

Data extraction was conducted independently by two reviewers (AJN and MA) to ensure accuracy and minimize bias. First, articles were filtered by title to exclude those that were not relevant. Examining the abstracts to determine their potential use was the next stage. In the end, full-text publications were assessed for appropriateness using the inclusion and exclusion criteria. Each stage included a discussion and resolution of any disputes among the reviewers. This methodical approach sought to improve the reliability and consistency of the research choices.

Results

The Google Scholar search yielded 897 entries, of which 42 studies published between 2020 and 2025 were considered relevant. The PubMed search yielded 32 papers published between 2015 and 2025; 28 met the eligibility criteria and were included in this review.

Mechanistic insights into the FLASH effect

Oxygen Depletion Hypothesis in FLASH-RT

**Figure 2:**
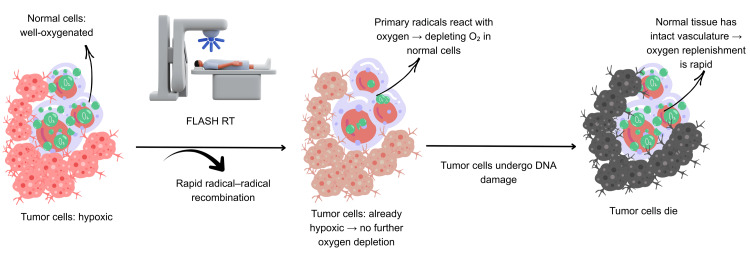
Mechanism of FLASH-RT. FLASH-RT rapidly depletes oxygen in healthy cells, creating transient hypoxia that protects them from DNA damage, while hypoxic tumor cells sustain irreversible damage and die. Credit: The image was created by the first author, Mariah Abraham. RT: radiotherapy

The concept of tumor hypoxia in RT was initially put out by Gottwald Schwarz in 1909, and LH Gray further confirmed this in the 1950s by showing that radioresistance is caused by reduced oxygen transport in malignancies [26]. The foundations of studies aimed at understanding the molecular basis of the FLASH effect have been expanded upon by several researchers. The oxygen depletion theory remains one of the most widely accepted hypotheses. It suggests that rapid molecular oxygen depletion induced by UHDR irradiation results in temporary hypoxia in normal tissues. This short drop in oxygen levels reduces indirect DNA damage and reactive oxygen species (ROS) production (Figure 2). ROS are efficiently neutralized by endogenous antioxidant systems, including glutathione (GSH), catalase, and superoxide dismutase (SOD) in healthy tissues. On the other hand, tumor cells are more susceptible to radiation-induced damage since they have a lower antioxidant capacity and higher oxidative stress. Healthy tissues are preferentially protected while tumor control is maintained by this special redox balance [37,38].

Free Radical Hypothesis

Transient oxygen depletion alone cannot account for the FLASH effect [39]. There are other biological mechanisms, including variations in redox metabolism, ROS signaling, and stress response pathways, which contribute significantly to the FLASH effect, rendering it more intricate and multifactorial than the oxygen depletion model indicates [40]. Superoxide is created when the peroxyl radical (ROO∙) is oxidized during FLASH-RT. A portion of this superoxide reacts with oxygen, forming hydrogen peroxide (H₂O₂). Ferrous iron (Fe²⁺) is released from redox-active proteins by another part. Under UHDR conditions, cancer cells produce more ROS because they have significantly higher levels of Fe²⁺ and transferrin receptors than normal cells. This Fe²⁺ promotes powerful Fenton reactions with H₂O₂ [41].

While total oxygen deprivation is unlikely, diminished reoxygenation and ROS production together account for the preservation of normal tissue observed in FLASH-RT [42]. Computational models and Monte Carlo (MC) simulations indicate that UHDR irradiation generates dense ionization clusters, which facilitate radical-radical recombination, hence reducing the production of harmful ROS and subsequent DNA damage in healthy cells. Conversely, other studies suggest that greater hydroxyl radical (∙OH) interactions are probably the source of increased H₂O₂ concentrations. Simulations generally indicate that FLASH may result in smaller, less diffuse ROS clusters and transient hypoxia that may not harm healthy cells. These simulations offer mechanistic insights, but their accuracy is constrained by assumptions of homogeneous solutions and the absence of biological complexity [41].

Immune Protection Hypothesis

The immune protection concept encompasses immunological and inflammatory responses that contribute to the normal tissue sparing in FLASH-RT [43-45]. The activation of immune effector cells, such as neutrophils, macrophages, and monocytes, which release proinflammatory cytokines, including tumor necrosis factor alpha (TNF-α), interleukin-1 (IL-1), and interleukin-6 (IL-6), is the first step in a robust inflammatory cascade triggered by radiation-induced lung damage. Through fibroblast activation and persistent transforming growth factor beta (TGF-β) signaling, this immunological activation causes secondary damage and fibrotic remodeling [46]. Increased TGF-β signaling decreases antitumor immunity by inhibiting the function of cytotoxic CD8⁺ T-cells and natural killer (NK) cells. In addition, it promotes polarization of M2 macrophages and expansion of regulatory T-cells, both of which reduce the efficacy of immune surveillance [47].

Through the cGAS-STING pathway, radiation triggers the immune system by releasing danger signals that activate regulatory T-cells and dendritic cells. By activating TGF-β and upregulating programmed death-ligand 1 (PD-L1) expression, radiation can also inhibit antitumor immunity. By binding to the T-cell receptor PD-1, PD-L1 sends a "stop" signal that reduces T-cell activity and enables tumor cells and other cells expressing PD-L1 to evade immune attack. Radiation may also directly destroy immune cells, further reducing the overall immunological response. FLASH-RT could circumvent this by restricting TGF-β induction, thereby strengthening the immune system's response to tumors [48]. Other possibilities put up to explain the FLASH effect include selective effects on cancer stem cells, transient myosin light chain (MLC) activation, and the relative sparing of circulating immune cells, all of which may influence tissue tolerance to UHDR [43]. Experimental studies have also shown that FLASH-RT dramatically lowers radiation-induced lung fibrosis by retaining alveolar progenitor cell numbers and minimizing chronic tissue remodeling. FLASH exposure inhibits TGF-β signaling and inflammatory cell influx, including Ccrl2⁺ neutrophils, leading to a regenerative and less fibrotic microenvironment [30].

Recent evidence also shows that FLASH-RT mitigates radiation-induced fibrosis by inhibiting the epithelial-mesenchymal transition (EMT). FLASH-RT's ability to preserve normal tissue is further elucidated by its restriction of EMT signaling and fibrotic remodeling, in contrast to CONV-RT, which activates TGF-β/SMAD and related pathways [49]. In vitro research on human bronchial epithelial cells has shown that FLASH-RT markedly reduces the expression of fibrotic markers, including vimentin, compared with CONV-RT. This indicates that FLASH disrupts the molecular cascade responsible for persistent lung fibrosis [25].

DNA Integrity Hypothesis

FLASH-RT keeps normal tissues stable by reducing complex DNA damage and preserving molecular integrity. The UHDR minimizes the number of clustered double-strand breaks (DSBs), thereby improving DNA repair. This helps protect healthy tissue and prevents lethal chromosomal alterations [50].

After radiation exposure, DNA repair proteins such as ATM, 53BP1, and γH2AX rapidly aggregate at DSB sites, generating radiation-induced foci (RIF) that activate chromatin remodeling and coordinate repair [51]. High linear energy transfer (LET) radiation leads to more complex DSBs, which persist longer and are more difficult for the cell to repair. When these fractures go unrepaired or are misrepaired, they cause micronuclei, chromosomal abnormalities, and loss of clonogenic survival, eventually leading to cell death or senescence in tumor cells. In subsequent phases, radiation causes broader transcriptional changes, including changes in mRNA levels and non-coding RNA regulation [52]. FLASH-RT is considered to restrict the persistence of complex RIF in normal tissues, contributing to chromatin stability [51].

There is experimental evidence that UHDR irradiation reduces oxidative base lesions and single-strand breaks (SSBs) while maintaining similar levels of DSBs compared to standard dose rates [53]. Genomic integrity in normal tissues is maintained by FLASH-RT, which prevents DNA radicals from forming adducts with oxygen and promotes transient hypoxia-mediated chemical repair [54]. Although clinical confirmation is pending, preclinical studies suggest that FLASH-RT reduces radiation-induced lung damage, such as fibrosis and pneumonitis, compared with CONV-RT. These protective advantages are linked to improved preservation of lung tissue architecture and decreased oxidative stress and inflammation [55].

Mitochondrial Hypothesis

**Figure 3:**
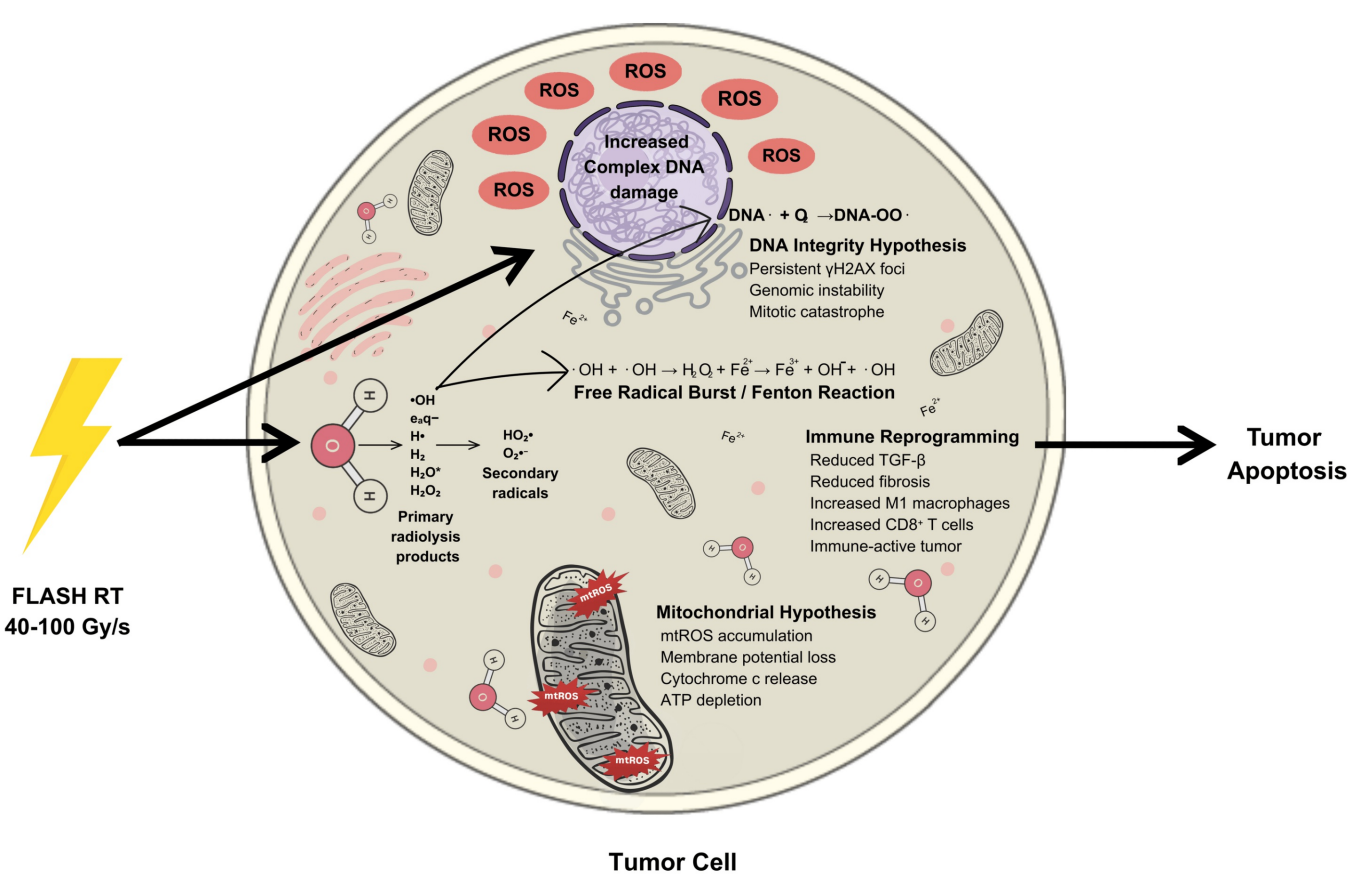
Mechanistic overview of FLASH-RT-induced tumor cell killing. FLASH-RT causes rapid water radiolysis, which produces ROS and iron-driven Fenton reactions that increase oxidative stress and DNA damage in tumor cells. It also increases mtROS, alters membrane potential, and promotes cytochrome-c release, which causes apoptosis. FLASH-RT reduces TGF-β signaling, boosting M1 macrophage polarization and CD8⁺ T-cell activity, resulting in stronger antitumor immunity and faster tumor cell killing. Credit: The image was created by the first author, Mariah Abraham. RT: radiotherapy; ROS: reactive oxygen species; mtROS: mitochondrial reactive oxygen species; TGF-β: transforming growth factor beta; ATP: adenosine triphosphate

Mitochondria are essential organelles that provide cellular energy via oxidative phosphorylation. To maintain cellular balance, cells also regulate key functions, including metabolism, autophagy, and programmed cell death [56]. Mitochondria are particularly vulnerable to oxidative damage when ROS surpass intrinsic antioxidant defenses [57]. In contrast to CONV-RT, FLASH-RT promotes mitochondrial function by lowering mitochondrial ROS (mtROS) levels, stabilizing mitochondrial proteins, and preventing apoptosis in healthy cells, which are oxidatively stressed and destroy mtDNA. In tumor cells, FLASH increases mtROS and mitochondrial dysfunction, sustaining tumoricidal efficacy. This equilibrium supports the mitochondrial metabolism theory as a crucial mechanism of FLASH-RT's protection of normal tissue [31]. These physicochemical, metabolic, immunological, and radiobiological reactions work together to explain why FLASH-RT kills tumors differently and spares normal tissues. Figure 3 shows a brief schematic of these mechanisms in a tumor cell under the effect of FLASH-RT.

Transient oxygen depletion alone cannot account for the FLASH effect [39]. There are other biological mechanisms, including variations in redox metabolism, ROS signaling, and stress response pathways, which contribute significantly to the FLASH effect, rendering it more intricate and multifactorial than the oxygen depletion model indicates [40]. Superoxide is created when the ROO∙ is oxidized during FLASH-RT. A portion of this superoxide reacts with oxygen, forming H₂O₂. Fe²⁺ is released from redox-active proteins by another part. Under UHDR conditions, cancer cells produce more ROS because they have significantly higher levels of Fe²⁺ and transferrin receptors than normal cells. This Fe²⁺ promotes powerful Fenton reactions with H₂O₂ [41].

While total oxygen deprivation is unlikely, diminished reoxygenation and ROS production together account for the preservation of normal tissue observed in FLASH-RT [42]. Computational models and MC simulations indicate that UHDR irradiation generates dense ionization clusters, which facilitate radical-radical recombination, hence reducing the production of harmful ROS and subsequent DNA damage in healthy cells. Conversely, other studies suggest that greater ∙OH interactions are probably the source of increased H₂O₂ concentrations. Simulations generally indicate that FLASH may result in smaller, less diffuse ROS clusters and transient hypoxia that may not harm healthy cells. These simulations offer mechanistic insights, but their accuracy is constrained by assumptions of homogeneous solutions and the absence of biological complexity [41].

Metabolic and Immunologic Reprogramming

Proton-based FLASH-RT alters metabolic pathways involved in energy metabolism and antioxidant defense, reducing the accumulation of chemicals associated with oxidative stress, according to comparative metabolomic analyses of human lung tissue. In these findings, FLASH may induce protective metabolic alterations that reduce radiation-induced oxidative damage [58]. Taken together, these metabolic and molecular changes explain how FLASH maintains tumoricidal efficacy while protecting healthy tissue. In a study, Ni et al. demonstrated that FLASH proton radiation reprograms tumor-associated macrophages to a proinflammatory state, thereby enhancing T-cell infiltration and activity in a medulloblastoma mouse model. FLASH reduces immunosuppressive macrophage polarization by reducing the expression of PPARγ, a lipid-regulating transcription factor that drives M2-like anti-inflammatory programs, and arginase-1, an enzyme that depletes arginine and suppresses T-cell proliferation. FLASH stops macrophages from entering an immunosuppressive M2-like state via decreasing PPARγ and arginase-1, as well as reducing lipid oxidation. This metabolic reprogramming promotes a more proinflammatory, tumor-fighting microenvironment. It improves CAR-T-cell efficacy, implying that modulating macrophage lipid breakdown is an important component of the FLASH-mediated immune response [59].

There is growing evidence that FLASH-RT reduces chronic inflammation and promotes tumor immunogenicity. This is achieved by modulating immunological responses and inducing simpler DNA DSBs. A solid biological foundation supports the clinical translation of lung cancer therapy. This is because these systems work together to prevent cancer and protect healthy tissues.

Linking Biological and Physicochemical Processes

Biological effects of FLASH-RT are caused by a series of extremely quick physicochemical reactions that start femtoseconds after irradiation. Intracellular water is initially radiolyzed, during which ionizing tracks deposit energy and produce primary species, including hydrated electrons, hydrogen atoms, ∙OH, and molecular byproducts such as H₂ and H₂O₂. At standard dose rates, these radicals typically migrate toward DNA and biomolecules. However, ionization events densely overlap under UHDR circumstances. As an alternative, two ROO∙ can recombine to form stable, non-radical products, reducing the total amount of harmful ROS before they can affect biological targets. According to computational simulations, oxidative yield in normal tissues is suppressed by recombination rates that are up to an order of magnitude greater under FLASH circumstances. A second physicochemical effect of FLASH is radiolytic oxygen depletion (ROD), which occurs when oxygen is rapidly consumed by radiation-generated electrons and hydrogen atoms, temporarily lowering tissue oxygen content during the FLASH pulse [37]. Even though recent research shows that oxygen is not completely depleted, it is believed that the brief hypoxia helps preserve normal tissue, particularly in tissues with adequate oxygen.

This is consistent with experimental findings that normal tissues, which are abundant in catalase and antioxidant capacity, detoxify H₂O₂ and organic peroxides more effectively than malignancies [11], whereas increased iron pools in tumor cells intensify oxidative damage and Fenton chemistry [60]. Beyond primary radical chemistry, FLASH-RT alters lipid peroxidation kinetics, as evidenced by studies showing linear peroxidation under CONV-RT and no significant lipid oxidation by UHDR in micelles and liposomes. This shows that radical recombination shortens redox chain reactions, reducing membrane damage in normal tissue. In contrast, tumor cells with defective antioxidant defenses accumulate more lipid-derived reactive species and organic peroxides under equivalent exposures, driving iron-dependent oxidative damage and potentially leading to ferroptosis. At the biological level, FLASH-RT influences mitochondrial function, a critical determinant of cell fate. Mitochondria produce adenosine triphosphate (ATP) and ROS via oxidative phosphorylation. However, excessive radiation-induced ROS leads to mitochondrial permeabilization, cytochrome-c release, and apoptosis. Conventional dosage rates cause significant mtROS and hinder electron transport, while FLASH-RT has been demonstrated to preserve mitochondrial integrity and minimize acute inflammatory responses in normal tissues [60]. Tumor cells, which are already undergoing metabolic reprogramming and producing higher baseline ROS, are more vulnerable to mitochondrial breakdown and oxidative death under UHDR conditions.

**Figure 4:**
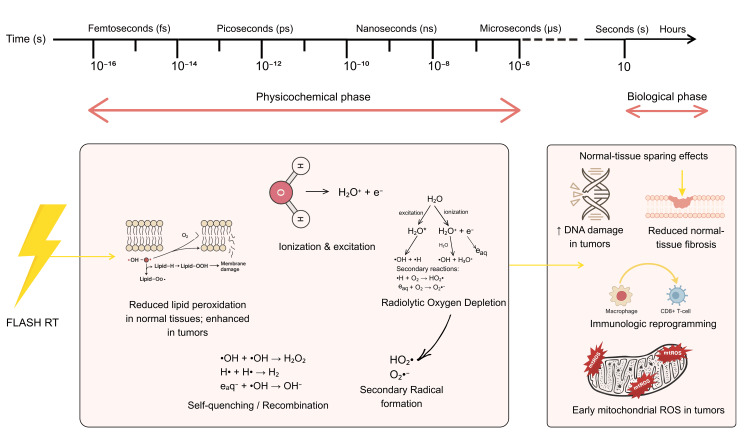
Physicochemical and biological mechanisms behind the FLASH-RT effect. FLASH-RT rapidly ionizes water, forming radicals that temporarily deplete oxygen. Normal cells quickly neutralize these radicals, limiting oxidative damage, but tumors store mitochondrial ROS and cause complex DNA damage. These physicochemical changes result in reduced fibrosis, enhanced immune activation, and selective tumor cell death. Credit: The image was created by the first author, Mariah Abraham. RT: radiotherapy; ROS: reactive oxygen species; mtROS: mitochondrial reactive oxygen species

These physicochemical reactions involving radical recombination, changed oxygen kinetics, suppressed lipid peroxidation, and differential mitochondrial responses work together to provide a coordinated biological outcome, as shown in Figure 4. Here, normal tissues are spared while tumoricidal efficiency is preserved. This multifactorial mechanism is supported by modeling studies on water radiolysis, redox dynamics, and oxygen transport, as well as experimental data from human tissues and in vitro settings. However, the therapeutic application of FLASH-RT requires a deeper understanding of the physicochemical and biological mechanisms underlying the FLASH effect [61].

FLASH-RT's radiation sources and delivery methods

**Figure 5:**
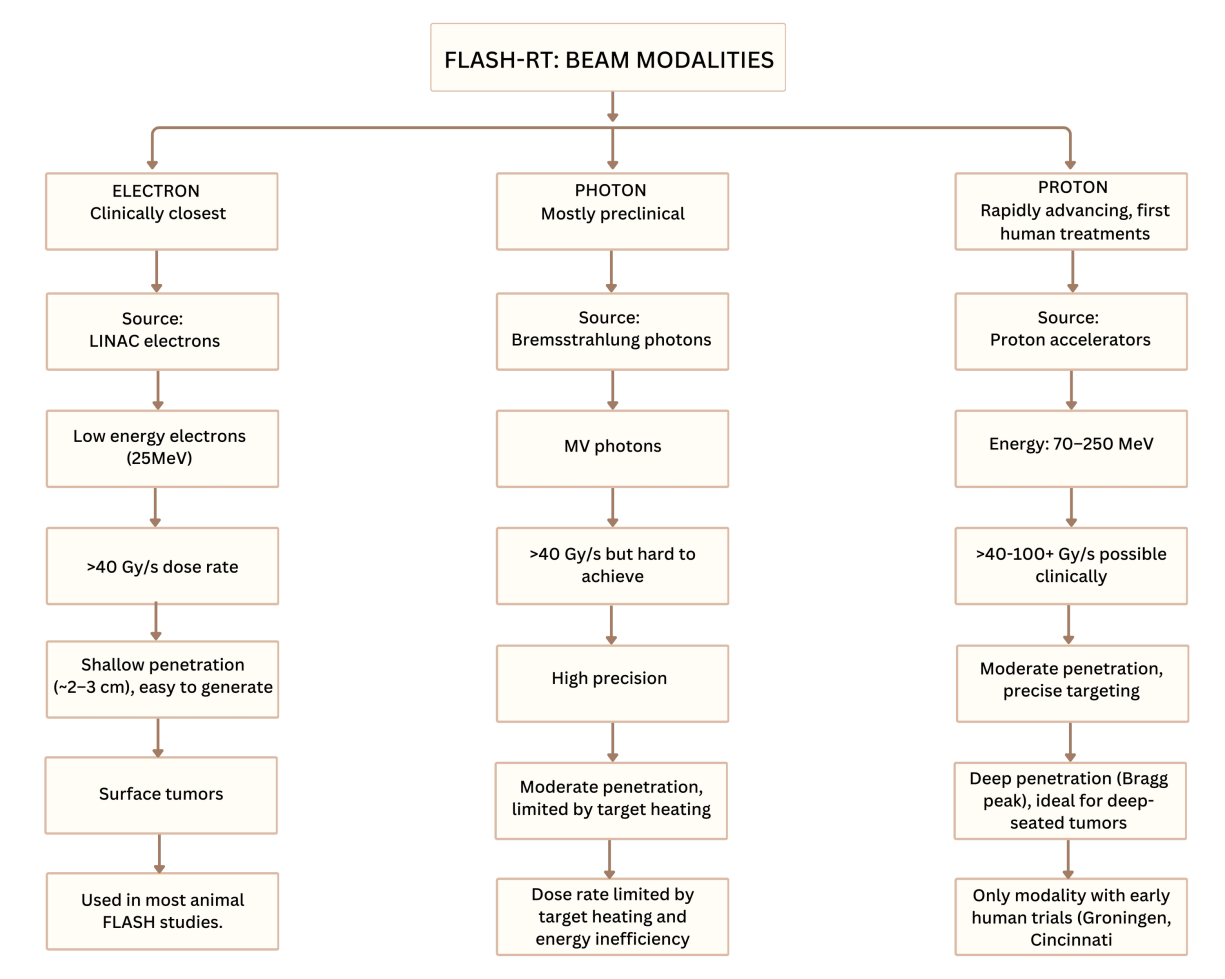
Overview of the FLASH-RT beam modalities. Electron FLASH is the most established and works best for shallow, surface tumors. Proton FLASH enables deep-seated targeting via the Bragg peak and is the only modality tested in early human trials. Photon FLASH remains mostly preclinical due to difficulties achieving ultra-high dose rates and managing heating. Credit: The image was created by the first author, Mariah Abraham. RT: radiotherapy; linac: linear accelerator

Protons, photons, electrons, or heavier ions can all be used as radiation sources in FLASH-RT. All of these have special biological and physical benefits. Depending on the depth of penetration, beam quality, and the pace of dose delivery, each beam type experiences the FLASH effect differently. This classification, as mentioned, is demonstrated in Figure 5.

FLASH Based on Electrons

Low-energy electrons (about 25 MeV) from experimental or therapeutic linacs were employed in the early studies on FLASH-RT [33]. Electron FLASH (eFLASH) efficiently controls cancers by delivering high radiation rates (>40 Gy/s) without damaging healthy tissue. According to research, it lessens toxicity in both in vitro and in vivo settings in normal tissue [18]. The US Nuclear Regulatory Commission (USNRC) safety standard of 20 µSv/h is exceeded by eFLASH-RT, which produces radiation leakage of around 10 µSv/h in regulated regions and 780 µSv/h in uncontrolled zones at 16 MeV and a 180° gantry angle. This highlights the necessity for enhanced shielding and safety measures in experimental FLASH-RT deployments [62]. While very high-energy electron (VHEE) FLASH may be able to treat deep-seated cancers, including lung tumors, low-energy eFLASH is effective and commonly used for treating surface cancers. Advancements in dosimetry and accelerator architecture support its therapeutic promise. Research has demonstrated that it preserves the tumor response in thoracic models and is less detrimental to the lungs [63]. The FLASH effect has been successfully demonstrated in several animal models and organs, even though beam uniformity and precise dosimetry for clinical translation remain challenges [64].

FLASH With Photons (X-rays)

Photon-based FLASH-RT preserves healthy tissue by using X-rays at high dose rates (≥40 Gy/s) and brief durations (<200 ms). It illustrates how challenging it is to reach such high rates when X-ray objects overheat and have inefficient energy transmission. Photon-based FLASH is caused by bremsstrahlung X-rays (photons), which are created when high-energy electrons collide with a tungsten target. Studies using synchrotron and bremsstrahlung systems have shown decreased lung damage in animal models, while preserving tumor control [65,66]. Improved thermoacoustic imaging signal-to-noise ratio allows for deeper and more precise dosage assessments with photon FLASH [34]. It is harder to test than electron or proton FLASH owing to UHDR issues. While photon beams may theoretically attain FLASH-level dosage rates, current linacs and monitoring systems cannot effectively measure or deliver such brief, intense pulses. Precision beam monitoring for photon FLASH applications requires improved dosimetry devices, notably rapid, radiation-hard detectors like thin silicon sensors [67]. Despite the potential for faster treatment and fewer side effects, photon-based FLASH-X-ray irradiation at very high speeds is still in the preclinical research stages due to technological limitations.

Proton-Based FLASH

Proton beams, compared with electron and photon FLASH, penetrate deeper, making them ideal for clinical translation [68]. It also offers better dose precision than electrons or photons [69]. It delivers radiation at very high rates, typically above 40 Gy/s. This creates the FLASH effect, which reduces the toxicity to normal tissue without compromising tumor control. Since protons can penetrate deeply into the body, they can treat both surface and deep-seated malignancies. This technology combines FLASH's biological benefits with high spatial accuracy, providing a viable approach to treating deep-seated tumors, such as those in the lung, where preserving healthy tissue is particularly important [70]. In NSCLC rodents, FLASH proton therapy (>60 Gy/s) exhibited a more robust tumor suppression than conventional proton therapy (<0.05 Gy/s). It resulted in increased antitumor immunity and reduced immune tolerance by reducing T-cells, shifting macrophages toward the M1 (antitumor) phenotype, and increasing tumor cell death with reduced proliferation. It also promoted CD8⁺ T-cells [18].

Proton FLASH has moved into clinical use, and 2019 was an important year because Groningen reached 200 Gy/s irradiation, and preclinical tests at Maryland revealed a 25%-30% drop in lung damage [71]. Proton FLASH-RT exploits the Bragg peak to target tumors with high accuracy while causing minimal harm to healthy tissue. In preclinical trials, it has shown lower toxicity and less fibrosis while still controlling tumors [9]. A new optimization framework, simultaneous dose and dose rate optimization (SDDRO), has been developed further to exploit the therapeutic potential of FLASH proton therapy. This method explicitly integrates dose rate limitations into the planning algorithm, in contrast to conventional dose-only optimization in intensity-modulated proton therapy (IMPT). SDDRO has the potential to optimize normal tissue protection by enhanced FLASH-dose rate delivery, as seen by its application in lung cancer models, which showed a notable increase in dose rate coverage (≥40 Gy/s) and higher dose rate uniformity [72].

Deep-Seated Tumor Applications

FLASH-RT is being expanded for deep-seated tumors, such as those in the thorax, although it remains technically challenging because UHDR irradiation requires a short delivery time. To overcome this issue, new systems are looking into megavoltage photon, transmission proton, ion, and VHEE (50-250 MeV) beams. VHEE-based 3D-conformal RT with a modest number of fixed broad beams provides a favorable balance between technical feasibility and dose requirements [73]. The exploration of transmission proton and MV-photon FLASH combinations for deep-target delivery while retaining normal tissue sparing is also helping to accelerate the translation of FLASH-RT into clinical use in deep-seated malignancies [74]. Proton transmission FLASH techniques are also rapidly developing. Proton transmission FLASH provides better normal tissue sparing and consistent dose coverage for deep targets than traditional Bragg peak administration because the dose is distributed outside the tumor to preserve UHDR throughout the target volume [75]. Recent preclinical and modeling studies, however, have demonstrated the feasibility of proton-based FLASH-RT and 3D-conformal VHEE, suggesting that deep-seated cancers may benefit from short-duration, normal-tissue-sparing radiation. Continued advances in biology modeling, dosimetry, and accelerator technology will be critical for safe and successful clinical translation.

Radiobiological principles

The FLASH effect is a phenomenon that has been shown to preserve normal tissue while still controlling tumors [76]. This phenomenon results from the interaction of physical, chemical, and biological systems, including oxygen dynamics, free radical interactions, vascular and immunological regulation, and tissue repair processes [13]. Durante et al. placed the FLASH effect within classical radiobiology through the dose rate effectiveness factor, demonstrating that rapid energy deposition at UHDRs diminishes intertrack lesion interactions, thereby lowering normal tissue toxicity without compromising tumoricidal efficacy [77]. The translation of preclinical evidence into clinical practice remains limited by an incomplete understanding of beam physics, dosimetry, and radiobiological response, despite robust preclinical results. Therefore, before extensive clinical application, it is imperative to develop high-fluence delivery systems and precise real-time dosimetry [78].

Technical and Dosimetric Aspects

Technically, it is challenging to obtain and maintain FLASH dose rates in thoracic applications. Proton and VHEE beams have emerged as viable options because they can give large dosage rates while maintaining acceptable penetration depth for lung cancer. Compared with intensity-modulated radiation therapy (IMRT), an advanced external beam RT technique, VHEE beams with energies between 100 and 200 MeV can achieve dose-averaged dose rates (DADRs) above 40 Gy/s [79,80]. Simulations show that maintaining an electron beam intensity above 9.3 × 10¹¹ e⁻/s enables FLASH-effective doses to cover over 90% of the target volume. Achieving dosage uniformity, reducing range uncertainty, and accounting for respiratory motion during administration remain tough issues [80].

Optimal tissue sparing is usually achieved when both the fractional dose (3.5-7 Gy) and the dose rate (>40 Gy/s) requirements are met. A single-beam-per-fraction (SBPF) configuration in stereotactic proton therapy obtains FLASH-eligible conditions. Still, it restricts conventional fractionation flexibility, necessitating a FLASH enhancement ratio (FER) of approximately 1.3 to maintain therapeutic benefit [68]. Therefore, it is imperative to balance biological safety, beam stability, and dose precision to achieve clinical success [76]. Conventional detectors saturate or under-respond under UHDR conditions [81]. Innovations include enhanced ionization chambers, diamond detectors for proton beams, and metal-oxide-semiconductor field-effect transistor (MOSFET) sensors for photons, which enable millisecond response time and real-time readout [82-85]. Despite these developments, the sector lacks standardized UHDR dosimetry techniques, emphasizing the need for verified, repeatable calibration frameworks.

Motion and Beam Optimization

As FLASH-RT approaches clinical use in thoracic oncology, it is critical to understand how motion and beam delivery restrictions interact with UHDR mechanisms. High-energy proton transmission beams (TBs) and VHEE beams deliver FLASH-compatible dose rates with acceptable target coverage, demonstrating conformity similar to or better than IMRT [38,86]. Despite these gains, respiratory motion remains a significant therapeutic limitation, as tumor displacement during ultra-short delivery times can reduce precision and impair dosage accuracy. Studies on respiratory-gated high-dose-rate radiation have reported reductions of up to 20% in mean lung dose and V20 compared with non-gated treatments; however, sustained beam delivery remains a concern [87]. Integrating motion management with enhanced proton and VHEE delivery systems may thus be critical to enabling safe and effective clinical application of FLASH-RT in lung cancer [73,76,87].

It remains challenging to translate FLASH-RT into clinical lung cancer treatment, despite promising preclinical and dosimetric evidence [30,80]. Significant technological improvements are needed to deliver UHDR to deep-seated thoracic targets in subsecond time frames. Consistent dosage delivery is made more difficult by patient-related variables such as tumor geometry and respiratory movements [80]. Furthermore, there is still no standardization of the optimal treatment parameters, such as fractionation, dosage limits, and biological endpoints. According to preliminary results, FLASH-RT's clinical value may be increased by combining it with motion management, sophisticated beam control, and maybe immunotherapy [30]. In the end, even though proton and VHEE FLASH-RT exhibit great translational promise, their safe and successful application in lung cancer treatment depends on resolving these biological and technical issues [30,80]. It is essential to have a solid understanding of these radiobiological concepts to optimize FLASH procedures and ensure reproducibility in clinical settings.

Data from translational, technological innovation, and preclinical research

Translational and Preclinical Findings

Vascular and microenvironmental effects: Mechanistic insights into the FLASH effect have progressively advanced through translational and preclinical investigations, underscoring its significant influence on the tumor microenvironment. Kim et al. examined FLASH-RT in Lewis lung carcinoma (LLC) xenografts and found that UHDR irradiation prevented the vascular collapse typically observed with CONV-RT. Standard exposure resulted in significant modifications in blood vessel morphology and elevated MLC phosphorylation, an indicator of vascular constriction and endothelial stress, but FLASH-RT maintained normal vessel integrity. This preservation of arterial patency promoted higher tissue perfusion and increased immune cell infiltration. It is noteworthy that MLC activation was determined to be ROS-independent and specific to CONV-RT, suggesting that the two modalities activate different molecular pathways. Furthermore, pharmacological reduction of MLC signaling in conventional treatment reproduced the protective vascular effects seen with FLASH, highlighting MLC-associated vascular dynamics as a viable translational target for radioprotection [88].

Pulmonary and neurological protection: FLASH-RT has been shown to significantly limit normal tissue toxicity, even at high doses, with studies demonstrating that doses exceeding 15 Gy reduced pulmonary fibrosis and preserved lung architecture while maintaining strong tumor control in mouse lung tumor models. FLASH-treated lungs have a largely intact alveolar structure and significantly lower collagen deposition than CONV-RT-treated lungs. Montay-Gruel et al. found that FLASH irradiation reduces oxidative stress and induces transient tissue hypoxia in the brain, a mechanism associated with reduced neuroinflammation and protection against radiation-induced cognitive impairment, implying that FLASH may have a broader systemic protective effect across both pulmonary and neurological tissues [89]. Collectively, these observations extend the protective capacity of FLASH beyond local tissue effects to include neurological and systemic domains, underscoring its potential to improve the therapeutic index of thoracic RT.

Effects on cellular function and dose rate: FLASH-RT maintains tumoricidal efficiency while significantly improving normal-cell survival, as demonstrated in cellular studies of A549 lung adenocarcinoma and normal bronchial epithelial cells. The FLASH effect was replicated in both single- and fractionated-dose regimens by irradiation at 200 Gy/s, thereby establishing 2 Gy as the minimal per-fraction threshold for sustained protection [58]. These results confirm the dose rate dependence of the phenomenon and imply that normal tissue sparing may be influenced by mechanisms other than transient oxygen depletion, such as differential DNA repair kinetics or ROS-mediated signaling [90]. Early clinical experiences also suggested that the method was potentially viable for translation. In elderly patients with thoracic malignancies, double-FLASH high-fraction radiation rapidly alleviated superior vena cava syndrome with acceptable normal tissue tolerance [91]. Together, these data suggest that FLASH-RT can retain tumor control while mitigating the toxicity traditionally associated with high-dose thoracic irradiation.

MC simulations and translational validation: The integration of laboratory findings with potential clinical applications has been significantly facilitated by precise dosimetric modeling. To optimize FLASH delivery, MC simulations have been implemented, which are essential for RT dose calculations. An innovative MC-based design for a clinical X-ray FLASH system has been demonstrated to achieve dose rates exceeding 40 Gy/s with conformal beam modulation, resulting in precise lung tumor coverage during a one-second exposure [21,92].

The landmark 2014 proof-of-concept trial offered the first substantial evidence by demonstrating that FLASH-RT (≥40 Gy/s, ≤500 ms) significantly diminished TGF-β-mediated fibrogenesis and protected against late pulmonary toxicity while preserving antitumor efficacy. TNF-α administration before irradiation reduced the protective benefit of the FLASH response, indicating an immune-mediated component [93]. FLASH-RT (200 Gy/s) and CONV-RT (0.033 Gy/s) were compared in subsequent validations using A549 xenograft and normal-lung models. The comparison was conducted across single-pulse (FLASH₁) and 10-pulse (FLASH₁₀) regimens. Both achieved tumor suppression; however, FLASH significantly reduced histologic lung injury, thereby confirming reproducibility across dose fractionation modes [90]. Collectively, these findings confirm that FLASH delivery preserves normal tissue integrity while maintaining tumoricidal activity, thereby establishing a solid preclinical foundation for technological translation.

Technological Advances in Proton FLASH

Rationale and biological evidence: FLASH-RT has been transformed from a conceptual framework into a clinically feasible modality through technological innovation [94]. Advances in adaptive treatment planning, intensity modulation, and image-guided RT have transformed and enhanced the administration of complex dosage accuracy and conformity while causing little injury to healthy tissues [86]. Despite these advances, lung cancer remains a serious problem because of its proximity to essential organs and the high radiosensitivity of pulmonary tissues. Traditional photon-based therapies often cause dose-limiting toxicities, including fibrosis, esophagitis, and pneumonitis [18]. The FLASH effect has been primarily investigated in biological systems using electron or photon beams. However, proton therapy offers an emerging platform for translating FLASH into clinical lung cancer management, owing to its reduced exit radiation and superior depth-dose profile [95].

Proton FLASH and immune response: Along with FLASH-RT, other next-generation RT modalities, such as proton-beam therapy, hold strong potential for more effectively preserving lymphocytes during treatment [96]. Proton therapy offers clear benefits in shielding surrounding tissues because it uses the Bragg peak to deposit the most energy within the tumor while limiting exit doses [18]. However, even proton treatment can cause late toxicities, especially if a large amount of healthy lung tissue is exposed or exposed to radiation [18,97]. Proton beams are particularly capable of reaching FLASH dosage rates when used with single-energy TBs that pass through the patient, utilizing the plateau region of the depth-dose curve rather than the Bragg peak. This eliminates the need for energy degraders in cyclotron-based systems and enables greater beam currents. Therefore, FLASH delivery can be carried out without requiring significant modifications to the proton treatment facilities currently in place. Crucially, TBs are less vulnerable to respiratory motion and range uncertainty, which is a vital aid in thoracic applications [97]. Collectively, these results indicate that proton-based FLASH-RT may broaden the therapeutic window by enhancing antitumor immunity while preserving healthy pulmonary structures.

TB feasibility: Recent research shows that single-energy proton TBs are now the most practical way to achieve FLASH dose rates without extensive beamline modifications, enabling very high beam currents by avoiding energy degraders. TB planning studies in lung stereotactic body RT (SBRT) have demonstrated stable dose delivery, reduced susceptibility to range errors, and better dose rate robustness as additional beam angles are utilized. Early clinical translation is ongoing, with studies such as FAST-01 using pencil beam scanning (PBS) TBs to deliver high-dose fractions safely in humans. Complementary translational research has shown that proton FLASH retains lung progenitor cells, lowers fibrosis, and maintains tumor control, indicating its suitability for thoracic applications [97]. Integrating these findings, current proton SBRT optimization frameworks provide additional evidence that TB FLASH can achieve clinically relevant dose rate coverage with excellent organ-at-risk (OAR) sparing, increasing its potential for real-world deployment [98].

Optimization and Emerging Engineering Strategies

Beam delivery and dosimetry: New types of radiation therapy, such as proton and carbon-ion RT (CIRT), image-guided RT, SBRT, and IMRT, provide more accurate targeting and higher doses. Apart from particle therapy benefiting from the Bragg peak, normal tissue toxicity and radiation-related side effects remain major issues in medicine, making life difficult for patients [99]. Among the known delivery options, high-energy TBs are presently the most practicable means of achieving proton FLASH conditions. However, they deliver a slightly higher integral dose than IMPT. Comparative planning evaluations show that although it produces the lowest OAR exposure, IMPT is presently unable to sustain FLASH dosage rates. TBs, on the other hand, frequently attain greater mean dose rates and comparable lung sparing, indicating a greater likelihood of a successful FLASH deployment [94]. These results highlight the need to reconcile dosimetric accuracy with the biological advantages of UHDR administration.

Advanced delivery systems: Advanced FLASH-RT delivery systems strive to achieve UHDR while maintaining high-quality dose conformity, a challenge for traditional linac-based platforms that cannot modify beams quickly enough to meet UHDR standards. The ROAD (ROtational direct Aperture optimization with a Decoupled ring-collimator) system solves this by combining a rapid slip-ring rotating linac with 75-150 preshaped multileaf collimator apertures, allowing for subsecond delivery at 76-112 Gy/s mean dose rates while retaining IMRT-level modulation. ROAD achieves complex dosage modulation and FLASH-compatible rates by eliminating mechanical leaf-motion delays and firing pulses only when the rotating source aligns with predefined apertures. Comparative results show greater sparing of OARs, lower integral dose, and enhanced planning target volume (PTV) uniformity compared with VMAT, indicating its viability as a next-generation FLASH delivery platform [38]. VMAT is a popular photon-based technology that delivers radiation in continuous arcs around the patient, making it the current clinical benchmark for high-quality dose conformity and treatment efficiency. Hence, it is used as a point of comparison. This method shows how radiobiological goals and mechanical design may be coupled to deliver safe and efficient treatments.

Hybrid and optimized beam solutions: Hybrid techniques that combine FLASH with CIRT are another new area of research. They provide excellent local control and very low toxicity in NSCLC, especially in patients with interstitial lung disease [100]. Complementary innovations, such as proton microbeam radiation (MRT), merge spatial fractionation with FLASH dose rates; in rat lung studies, MRT reduced fibrosis and enhanced tissue preservation relative to broad-beam FLASH [101]. Likewise, accurate Bragg peak delivery is enabled by proton PBS, which reduces the exit dose and protects surrounding tissue. Compared with TB approaches, Bragg peak FLASH reduced lung radiation exposure (V7 Gy and V7.4 Gy) by up to 40%, according to studies on lung cancer treatment planning. This improvement was further enhanced by optimizing beam angles [102]. Optimization of dose fractionation is also a critical factor. The comparative planning of uniform-fractional-dose (UFD) and non-UFD protocols demonstrated that the synergistic effects of dose fractionation and the FLASH mechanism were highlighted by enhanced normal tissue protection without compromising tumor coverage through the customization of fractional doses (5-24 Gy) [103]. These developments suggest that the therapeutic window of FLASH-RT can be further refined by meticulously adjusting both biological parameters and physical delivery.

Integrated modalities and microbeam approaches: To maximize delivery effectiveness, a genetic-algorithm (GA)-based optimization approach has been proposed for sequencing proton-beam locations during PBS FLASH. Simulations indicated that GA optimization enhanced UHDR coverage (V40 Gy RBE/s) of both tumor targets and OAR by 30%-40% without compromising the integrity of the plan [104]. Simultaneously, multienergy Bragg peak FLASH techniques combined with large-momentum-acceptance superconducting (LMA-SC) gantries and universal range shifters (URS) have significantly increased beam-switching speed and energy efficiency. Compared to TB systems, Bragg peak-based FLASH reduced the mean normal tissue doses and monitor unit (MU) requirements while maintaining full UHDR coverage [105]. Even with these rapid advances and developments, there are still significant obstacles to overcome before FLASH-RT is widely applied to patients with lung cancer. Maintaining constant UHDR across large thoracic volumes and ensuring precise dosimetry during proton pencil beam delivery are two major technological problems [102]. FLASH-RT was investigated in human lung precision-cut slices using integrated UHDR electron irradiation in combination with systemic agents such as carboplatin and docetaxel to assess potential synergistic or antagonistic interactions. The microscale precision-cut lung slice (PCLS) platform enables controlled assessment of viability, proliferation, and tissue-level injury, distinguishing between FLASH and conventional dose rate responses [106]. High-resolution microdosimetry with a modified Gafchromic EBT-XD film also revealed precise spatial dosage distribution. This customized film, calibrated for UHDR settings, enables fine-scale mapping of radiation-deposit locations within the target. By measuring dosage changes at the micrometer level, FLASH beams can be accurately verified as delivering the target dose uniformly and consistently across the irradiation field [101].

Emerging Therapeutic Perspectives: FLASH-RT in Pleuropulmonary Blastoma

Pleuropulmonary blastoma (PPB) is a rare primary malignant lung tumor of infancy. It accounts for only 0.5%-1% of all primary malignant lung tumors and is one of the three main subtypes of pediatric pulmonary malignancies, along with fetal adenocarcinoma and pulmonary blastoma [107]. Although rare, multimodal treatment can cure patients with relapsed disease of PPB type II/III [108]. It is a very uncommon pediatric cancer with a dismal prognosis and significant biological heterogeneity. The challenges in identifying and treating PPB type 2 were highlighted by a case report presenting as a right lung abscess. Given PPB's aggressive nature and our limited knowledge of its molecular causes, modern treatment techniques are increasingly resorting to cutting-edge modalities, including proton therapy and FLASH-RT. Despite the paucity of preclinical and clinical evidence for PPB, the inclusion of FLASH-RT as a potential future option reflects growing interest in leveraging its radiobiological advantages to improve local control while reducing collateral harm to developing tissues. The combination of genetic testing, oncological counseling, and AI-driven radiomics may serve as a roadmap for future translational research in rare illnesses, potentially enhancing diagnostic precision and therapeutic customization [109].

Ridge Filter-Based Integrated Optimization Framework for Proton FLASH

Research introduced a new enhancement framework that combines patient-centered ridge filters and IMPT planning to satisfy the unique requirements of UHDR FLASH delivery. Dosage, DADR, and dose-averaged LET (LETd) were all simultaneously altered using the integrated physical optimization (IPO-IMPT) approach. By creating ridge filters for a 250 MeV fixed-energy proton beam using inverse planning software based on MC simulations, the method tailored the spread-out Bragg peak to each patient's lung tumor [110]. In three example lung cancer patients, the IPO-IMPT system yielded superior outcomes than regular IMPT. Specifically, it increased the dose rate, decreased the LET in OAR, and preserved target coverage. Sparse-optimized ridge filters, which eliminated certain pins, enabled higher dosage rates and faster administration than regularly spaced filters. The framework demonstrated that DADR and LETd adjustments together may enhance treatment effectiveness and normal tissue sparing while maintaining FLASH-compatible dose parameters [110].

Preclinical Evidence and Dose Rate Considerations for PBS FLASH in Lung Tissue

Translational and human tissue evidence: In animal models, exposure to UHDR protected progenitor cells and reduced pulmonary fibrosis, according to early research on the FLASH impact on lung tissue. Dubail et al. examined precisely cut lung slices from lobectomy patients to apply this to human systems. They discovered that samples susceptible to FLASH-RT had a significantly higher proliferative capacity than samples susceptible to conventional dose rates. About 95% of samples demonstrated normal tissue sparing. With only 55 genes differentially expressed compared to over 2,000 in CONV-RT, transcriptomic analyses also showed that FLASH decreased activation of oxidative stress pathways, p53-mediated apoptosis, and DNA damage checkpoints, indicating a diminished stress response in normal parenchyma. Interpatient variability, however, suggested that responsiveness to FLASH might be influenced by inherent biology [29].

FLASH-RT administered at UHDR has shown efficient tumor suppression and superior normal tissue sparing in preclinical species, including mice, minipigs, cats, zebrafish, and one human patient [23]. While early research predominantly used electron beams, more recent studies use photon and proton beams, with PBS providing exact target conformity, OAR sparing, and deep tumor therapy. Proton beams are the best kind of beam for FLASH because they can produce high beam currents. Furthermore, preclinical translation in biological research, treatment planning, and system design has improved when PBS and FLASH are combined. Among the specific issues with PBS FLASH planning are dose rate measurement, equipment constraints, and attaining enough OAR FLASH coverage [111]. Due to its precision, PBS-administered proton therapy is growing in popularity. Recent preclinical research indicates that PBS FLASH-RT can reduce normal tissue damage while preserving tumor control. This implies that it can translate. Nonetheless, a significant barrier to clinical application is the lack of agreement over the definition of dose rate in PBS distribution, which complicates cross-study comparisons and might affect the repeatability of clinical translation [112]. The disagreement over dose rate measurement in PBS remains a significant barrier to translation.

Quantitative framework for dose rate assessment: Dose rate measurement in proton PBS FLASH-RT is based on three unique metrics: DADR, averaged dose rate (ADR), and dose-threshold dose rate (DTDR), each of which captures different temporal and spatial aspects of spot delivery. DADR provides the best dose rate estimation by weighting contributions from all locations, regardless of dwell or scanning time, resulting in more uniform dose rate distributions. In contrast, ADR integrates spot-delivery duration and scanning time, yielding the lowest dose rates and demonstrating reliance on scanning direction. DTDR, which uses a fixed-dose threshold (0.1 Gy) to exclude low-dose spot tails, produces intermediate dose rates and emphasizes instantaneous contributions, which are crucial for biological correlations. All data show dose rate attenuation with depth, underscoring the importance of minimizing MUs/spot size for OAR coverage at ≥40 Gy/s. Higher MU considerably improves the achievement of FLASH rate [113].

Developments in Bragg peak FLASH planning and optimization: Recent studies have examined the feasibility of treating lung malignancies with this method by comparing the dosimetric performance of an advanced proton PBS Bragg peak FLASH approach with that of conventional PBS-based IMPT. Using this technology, single-energy PBS Bragg peak FLASH plans were generated with a novel Bragg peak tracking approach, enabling the Bragg peaks to stop precisely at the distal border of the target. These designs achieved adequate FLASH dosing rates and IMPT-equivalent dose compliance using multiple-field optimization (MFO) [35].

Building on the advancements in FLASH-RT, SBRT represents yet another significant technological advance in targeted radiation delivery. SBRT demonstrated comparable outcomes to surgical metastasectomy in lung cancer, establishing it as a reliable, less invasive treatment for pulmonary lesions and offering a strong technological foundation for the actual use of FLASH-RT [86]. Bragg peak FLASH designs fared better than conventional plans in terms of V40 Gy/s coverage, or the proportion of tissue getting UHDR, across all major OAR in a trial of 10 lung cancer patients treated with SBRT. Nearly all OAR reached FLASH dose rates at dose thresholds greater than 5 Gy, suggesting the potential for successful normal tissue sparing. Although IMPT demonstrated somewhat improved target-dose uniformity, there were no statistically significant differences between Bragg peak FLASH and CONV-RT for spinal cord, esophagus, heart, and lung gross tumor volumes (GTVs) [35]. To enhance normal tissue sparing, a recent study examined fractional dose optimization in Bragg peak FLASH-RT for lung cancer. Both UFD and non-UFD approaches were used to deliver 50 Gy in five fractions to 15 patients with central and peripheral cancers. Non-uniform dose planning reduced lung and heart doses by about 4% and 10%, respectively, while maintaining similar target coverage and improving OAR protection. Both approaches showed high dosage conformality, with no appreciable differences for the spinal cord or esophagus. According to these results, modifying fractional doses in Bragg peak FLASH-RT may improve normal tissue sparing by fusing the advantages of fractionation with the FLASH effect [20].

A recent study developed a Bragg peak FLASH planning technique that preserves UHDR while improving organ sparing by using range pull-back and inverse optimization. This method reduced the lung dosage by up to 32%, reduced unnecessary dose leakage in six cases of lung cancer, and achieved target coverage comparable to that of TB designs. The Bragg peak approach demonstrated that it could still deliver clinically viable plans with enhanced normal tissue protection, even though TBs provided far greater dose rate coverage [114].

Effective preclinical implementation and delivery: The dosage rate dispersion and delivery effectiveness of proton-based FLASH-RT in stereotactic lung cancer treatment were assessed in a preclinical investigation. To deliver UHDR to seven patients, 244 MeV proton TBs were generated with the Bragg peak outside the body. The metrics compared to VMAT were tissue coverage, dose rate distribution, irradiation duration, and plan quality. The results showed that proton FLASH designs offer as much or greater lung, thoracic wall, and heart sparing than VMAT. Approximately 40% and 75% of the total dosage, respectively, were administered at FLASH-effective levels for spot peak dose rates (SPDRs) of 100 and 360 Gy/s. Low-dose-rate contributions from periphery areas prevented full FLASH coverage. Approximately 85% of the irradiated body volume was exposed by one or two beams, with total beam delivery periods ranging from 300 to 730 ms. The study concluded that successful proton FLASH application requires adjusting dosimetric, temporal, and spatial parameters. FLASH efficiency is improved by reducing delivery time and increasing SPDR, providing a useful foundation for future clinical proton FLASH design [115]. Table 1 summarizes the entire data on translational, technological innovation, and preclinical research in FLASH-RT.

**Table 1:** Summary of data from translational, technological innovation, and preclinical research in FLASH-RT TB: transmission beam; RT: radiotherapy; IMPT: intensity-modulated proton therapy; CIRT: carbon-ion radiotherapy; NSCLC: non-small-cell lung cancer; MRT: microbeam radiation; PBS: pencil beam scanning; LMA-SC: large-momentum-acceptance superconducting; URS: universal range shifters; OAR: organ-at-risk; IPO-IMPT: integrated physical optimization-intensity-modulated proton therapy; DADR: dose-averaged dose rate; LETd: dose-averaged linear energy transfer; LET: linear energy transfer; ADR: averaged dose rate; MFO: multiple-field optimization; SBRT: stereotactic body radiotherapy; UHDR: ultra-high dose rate

Technique/study focus	Model/study type	Dose rate (Gy/s)	Key findings	Ref.
TB optimization	Planning comparison (IMPT vs. TB)	>40	TB achieved higher mean dose rates with comparable lung sparing vs. IMPT	[94]
ROAD (ROtational direct Aperture optimization with a Decoupled ring-collimator) system	Simulation study–lung cancer	~70	Ultra-fast treatment (<1 s) with superior conformity using slip-ring accelerator	[38]
Hybrid FLASH + CIRT/proton microbeam	Rat lung, NSCLC planning	40–100	MRT and Bragg peak FLASH reduced fibrosis and lung dose (↓ 40% V7 Gy)	[100-102]
Genetic-algorithm (GA) spot-sequence optimization	PBS simulation–lung	≥40	Enhanced V40 Gy RBE/s coverage by 30%–40% without loss of plan quality	[104]
LMA-SC gantry + URS for Bragg peak FLASH	Proton system design	>40	Faster energy switching; reduced OAR dose and monitor units	[105]
IPO-IMPT with ridge filters	3 lung cancer patients	>40	Optimized DADR + LETd; sparse filters increased dose rate and reduced LET	[110]
Human lung slice study (Dubail et al.)	Precision-cut human lung tissue	~100 (equiv.)	95% normal tissue sparing; reduced oxidative stress and DNA damage gene expression	[29]
Dose rate metric comparison (Kang et al.)	Hypofractionated lung plans	0.05–200	DADR most consistent; ADR underestimated due to scanning delays	[113]
Bragg peak FLASH planning (MFO & SBRT)	10 lung cancer patients	>40	Increased V40 Gy/s coverage; reduced lung and heart dose by 4%–10%	[20,35]
Inverse optimization with range pull-back	6 lung cancer cases	>40	Lowered mean lung dose by 32% while maintaining UHDR	[114]
Preclinical proton FLASH (244 MeV TBs)	7 SBRT-modeled patients	100–360	40%–75% dose at FLASH-effective rates; 300–730 ms delivery	[115]

Clinical implementation challenges in FLASH-RT for lung cancer

There has been significant progress made in preclinical and translational research. However, the clinical application of FLASH-RT in lung cancer continues to be complicated and involves a variety of different strategies. Dosimetric errors, technical limits, biological variability, and the absence of uniform treatment settings across facilities are the primary problems that need to be addressed [14,22,36].

Dosimetry and the Procedure of Standardizing Dose Rates

One of the most critical challenges to FLASH-RT's clinical translation is the reliable quantification, verification, and reproducibility of UHDRs. Currently, there is no single, universally acknowledged metric for defining the physiologically appropriate dose rate threshold required to trigger the FLASH effect. This disagreement arises because the FLASH effect is controlled not only by the average dosage rate but also by the instantaneous dose rate, pulse structure, pulse duration, and total delivery time [112,113]. As a result, many researchers use different criteria to determine if an irradiation procedure meets FLASH requirements. This heterogeneity leads to significant variation in determining whether target volumes and OAR regions meet FLASH requirements in comparative investigations of DADR, ADR, and DTDR [113]. As a result, even when nominal dose rates are reported at equal levels, conclusions about normal tissue sparing or tumor response can vary significantly. The lack of consistent dose rate definitions impedes cross-study repeatability, regulatory evaluation, and multicenter validation, all of which are required for clinical adoption.

Furthermore, real-time verification of dosage distribution at UHDRs remains severely limited. The majority of dosimeters now used in clinical radiation therapy were designed and calibrated for conventional dose rates and are thus unsuitable for FLASH conditions. Under UHDR exposure, these detectors often saturate, lose linearity, or exhibit delayed response, rendering them unreliable for accurate dose assessment [21,111]. These problems underscore the critical need for standardized dosimetric frameworks to characterize dose delivery in FLASH settings accurately.

Dosimetric Challenges and Reproducibility Issues in Preclinical FLASH Studies

Several technical limitations in preclinical FLASH research threaten the accuracy and reliability of experimental dosimetry. Published radiobiological results frequently show significant variability, with documented discrepancies ranging from 12% to 42%. This heterogeneity is primarily due to differences in beam intensity profiles, field geometry, and dose-definition parameters used across different experimental platforms. The majority of preclinical FLASH systems use modified clinical accelerators that were not developed for UHDR delivery. These systems often lack feedback-controlled beam modulation and specialist UHDR treatment-planning software, limiting the accuracy with which dosage delivery may be controlled. Furthermore, commonly used dosimetric detectors, such as ionization chambers and scintillators, are susceptible to saturation effects under FLASH conditions, thereby lowering measurement accuracy.

Variations in pulse structure, repetition frequency, and beam stability among experimental setups add to the uncertainty. Differences in calibration methods, detector temporal response characteristics, and phantom configurations cause systematic inaccuracies in dose reconstruction. Collectively, these constraints impede the assessment of biological endpoints, making it difficult to separate actual FLASH-specific biological effects from aberrations caused by dosimetric discrepancies [116]. As a result, repeatability across laboratories remains a significant unmet challenge in preclinical FLASH research.

Management of Motion in Thoracic Points of Interest

Due to its continual breathing motion, the lung is one of the most technically demanding anatomical sites for FLASH-RT administration. Tumor movement during breathing introduces spatial uncertainty, which can lead to misalignment between the anticipated and delivered dose distributions, especially during subsecond beam delivery. Such misalignment may jeopardize dosage compliance and target coverage [35,76,87]. Although respiratory-gated and 4D imaging-guided techniques have shown up to a 20% reduction in mean lung dose in CONV-RT, applying these gains to FLASH dose rates remains technically problematic [87]. FLASH delivery's ultra-fast nature limits the practicality of real-time motion compensation, gating synchronization, and adaptive correction. TB techniques have demonstrated relative resistance to motion-induced distortions and range uncertainty. Despite these strategies, achieving constant ultra-high dynamic ranges across moving anatomical structures remains difficult [97,102]. As a result, adequate motion management remains a significant unresolved issue for the safe and precise delivery of FLASH-RT to thoracic targets.

Limitations of the Technology and Equipment

The clinical application of FLASH-RT requires customized beamlines capable of delivering dose rates of at least 40 Gy/s while maintaining constant beam current and minimizing beam spill. Currently, only a small number of clinical proton facilities have the equipment to accommodate such beam characteristics [30,80]. The limited availability of FLASH-RT inhibits its widespread use among treatment centers. Machine-specific restrictions further restrict spatial and temporal dose homogeneity. Beam current modulation capabilities, gantry momentum acceptance, and scanning magnet responsiveness all directly impact dose conformity and delivery precision [105,114]. Engineering solutions, such as LMA-SC and ROAD optimization systems, are being developed to solve these problems by increasing beam efficiency and dosage compliance [38,105]. However, incorporating these technologies into typical clinical workflows necessitates significant infrastructure changes, rigorous quality assurance methods, and regulatory validation. These constraints provide a considerable obstacle to widespread clinical application.

Variability in Biological Factors and Between Patients

Despite growing experimental evidence supporting the FLASH effect, the precise biological mechanisms underlying it remain unknown. Possible possibilities include temporary oxygen depletion, modification of oxidative stress pathways, and immunological remodeling. Importantly, these pathways can vary widely across tissue types and among individual patients [13,25,38]. Dubail et al. found evidence of interpatient variation in lung tissue response to FLASH, suggesting that individual differences in oxygenation status, DNA repair capacity, and baseline oxidative metabolism may influence treatment outcomes [29]. Such diversity complicates patient selection and calls into question the premise of uniform normal tissue protection. Furthermore, it is unknown how diseases such as chronic obstructive pulmonary disease (COPD) or pulmonary fibrosis affect FLASH efficacy [30,31]. These circumstances can affect tissue oxygenation, inflammation, and repair kinetics, potentially changing both therapeutic benefit and toxicity risk. As a result, the development of predictive biomarkers and better patient categorization procedures may be required for successful clinical implementation.

Design of Clinical Trials, Safety, and Current Regulations

The use of FLASH-RT in human clinical trials necessitates careful evaluation of ethical, safety, and regulatory concerns. Currently, there is inadequate evidence on late toxicities, the risk of future malignancies, and the accuracy of dose reconstruction under UHDR settings [22,33]. These uncertainties are especially troubling considering the possibility of delayed radiation-induced consequences that may not be apparent for years after treatment. As a result, insufficient long-term safety evidence is a significant obstacle to regulatory approval, limiting the advancement of FLASH-RT from experimental to routine clinical use. Regulatory agencies require detailed evidence demonstrating both efficacy and safety, particularly when novel dose-delivery paradigms differ significantly from standard radiation methods.

In addition to biological uncertainty, technical and infrastructure constraints further impede clinical translation. The lack of commercially available, clinically grade UHDR beam-monitoring equipment and defined calibration procedures presents substantial obstacles to institutional approval and quality assurance. Accurate real-time dosimetry and beam verification are critical for patient safety, especially given the high dosage rates involved. Because of these limits, the majority of active clinical research is still in the early stages of feasibility, with studies focusing on surface tumors, brain lesions, and cutaneous malignancies rather than deep-seated lung cancers [14,22]. These indications enable simpler beam delivery and dose verification, lowering technical risk during early clinical evaluation.

Globally unified techniques for beam characterization, dosimetry, and biological endpoint assessment are urgently needed for FLASH-RT to be widely adopted in clinical practice. Such uniformity is essential for producing consistent data, facilitating regulatory evaluation, and assisting in the organization of multicenter clinical studies. Without uniform technical and biological frameworks, interpreting trial results would be difficult, and implementing FLASH-RT in clinical practice would encounter significant challenges.

Integration of Multimodal Therapies as a Treatment Option

Combining FLASH-RT with systemic drugs such as chemotherapy, targeted therapy, or immunotherapy has the potential for theoretical synergy, as these treatment modalities may interact to improve overall therapeutic outcomes; however, this approach introduces complex biological and logistical challenges that have yet to be resolved [25,30]. The interaction between UHDR irradiation and systemic medicines is not entirely understood, specifically how ultra-rapid dose administration affects immune signaling, treatment tolerance, and therapeutic component coordination. FLASH has been shown in preclinical models to have immunomodulatory effects, including increased CD8⁺ T-cell infiltration and decreased regulatory T-cell populations. This suggests potential interactions with immune-based therapies [18]. These findings suggest that FLASH-RT may impact the tumor immune environment in ways that complement systemic treatments. Nonetheless, converting these effects into a predictable clinical benefit is uncertain.

Despite these hopeful findings, the appropriate sequencing of FLASH-RT compared to systemic therapy has yet to be clinically verified. It is uncertain if FLASH-RT should be given before, during, or after systemic treatment to enhance efficacy and minimize side effects. Furthermore, the probability of radiosensitization in UHDR settings is uncertain, as biological responses at UHDR may differ from those at CONV-RT, thereby affecting expected therapeutic interactions. Conventional fractionation plans are usually intended to promote immunological recovery and normal tissue healing over time. These established scheduling rules may conflict with FLASH's ultra-rapid delivery paradigm, further complicating the incorporation of combination therapy techniques into existing clinical procedures.

Uncertainty in Long-Term Normal Tissue Toxicity and Functional Lung Preservation

Although FLASH-RT is expected to protect healthy lung tissue, new evidence suggests this advantage may not hold across all dose ranges. Preclinical results indicate that radiation pneumonitis and fibrotic remodeling can occur at higher doses, implying that FLASH's protective window may be dose-dependent rather than absolute. Importantly, functional assessments revealed that both FLASH and conventional dose rate irradiation can reduce tidal volume and residual lung capacity depending on the respiratory phase at the time of exposure, demonstrating that structural preservation does not always imply continued physiological performance. These findings reveal an important gap in understanding how FLASH affects long-term lung biomechanics, airway compliance, and gas exchange capacity. The uncertainty surrounding chronic toxicity, particularly beyond acute and subacute time points, makes it challenging to define safe therapeutic indices in lung cancer patients, many of whom already have compromised pulmonary reserve due to a smoking history, COPD, or tumor-induced obstruction [117]. As a result, implementing evidence-based dose-safety thresholds, establishing functional objectives, and long-term methods are critical to ensuring that clinical FLASH-RT planning strikes a compromise between tumor reduction and lung health maintenance.

Current Emphasis on Palliative and Low-Risk Indications Results in a Scarcity of Clinical Evidence in Curative Contexts

Most active FLASH-RT clinical trials are still focused on palliative or low-risk indications, such as single-fraction 8 Gy treatments for symptomatic non-spinal thoracic bone metastases. These studies are primarily intended to assess short-term safety, feasibility, and acute toxicity reduction, rather than long-term tumor control. While they provide helpful information on healthy tissue sparing and procedural tolerance, they do not produce evidence relevant to curative-intent RT, which requires greater doses, many fractions, and complex dose distributions. Furthermore, superficial or low-risk clinical models do not reflect the morphological, biochemical, and motion-related features of deep intrathoracic lung cancers. The lack of data on high-dose hypofractionation, tumor-microenvironment interactions, late toxicity, and long-term local control creates a significant translational gap. Before FLASH-RT may be used in ordinary curative practice, it must go through a planned transition from palliative indications to carefully monitored phase I/II trials in early-stage or locally advanced lung cancer [118].

Obstacles of an Economic and Logistical Nature

The expense and difficulty of constructing FLASH-capable infrastructure constitute a significant obstacle to the broad clinical implementation of FLASH. The commissioning of dosimetry devices for UHDR verification, the training of specialists, and the upgrading of existing proton treatment facilities all require significant financial investment [14,86]. Proton or high-energy linac installations are still prohibitively expensive for most centers. As a result, the availability of these services is restricted in low- and middle-income regions, where the incidence of lung cancer is the highest. To ensure worldwide fairness in access to treatment, this disparity underscores the need for affordable, scalable UHDR systems [119].

**Figure 6:**
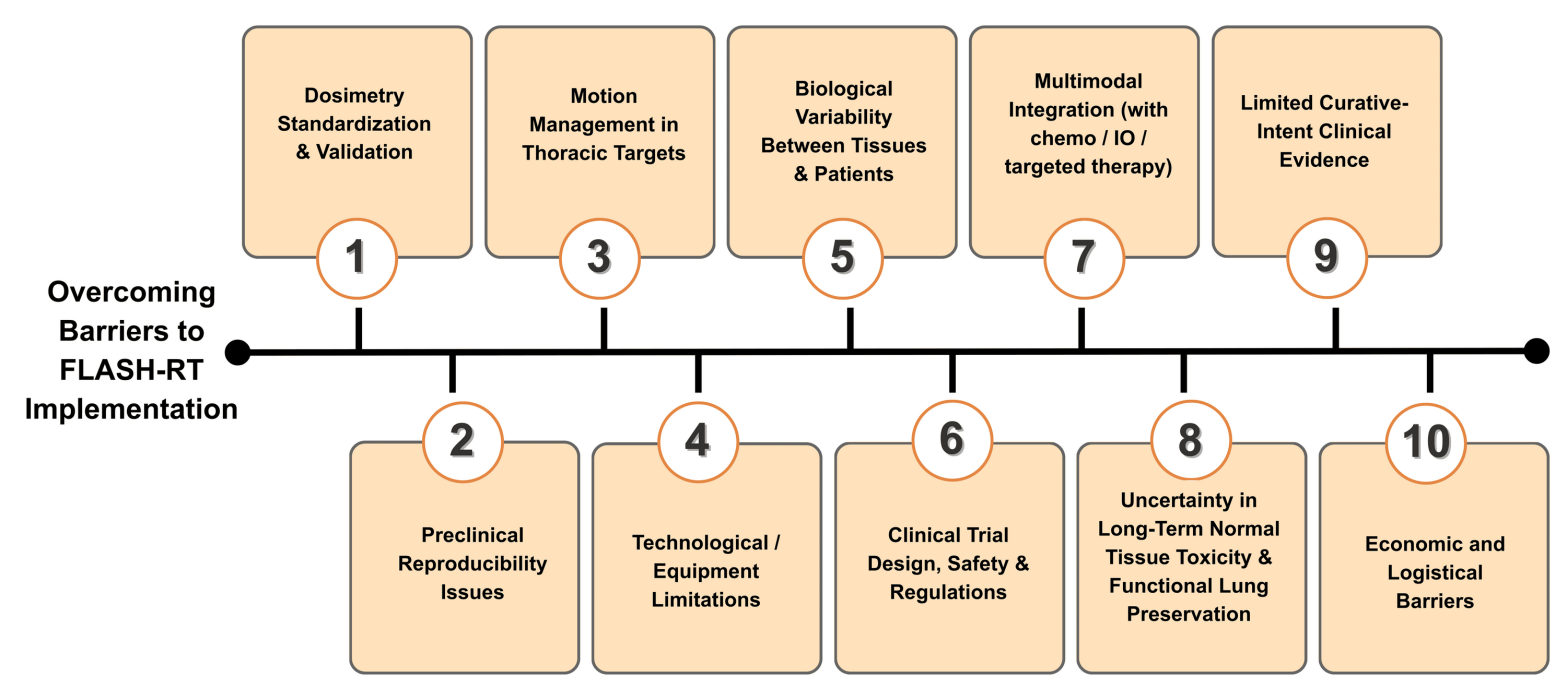
Summary of the major challenges in using FLASH-RT to treat lung cancer. Credit: The image was created by the first author, Mariah Abraham. RT: radiotherapy; IO: immunotherapy

In conclusion, although FLASH-RT has the potential to improve the therapeutic ratio in lung cancer significantly, its successful clinical use depends on overcoming operational, biological, and technical challenges, as summarized in Figure 6. The creation of standardized dosimetry frameworks, enhanced motion correction systems, and multicenter clinical trials are among the next steps required to close the gap between the promise of the lab and the realities of clinical practice [22,33,36].

## Conclusions

FLASH-RT has emerged as a breakthrough technique in thoracic cancer, exhibiting the potential to retain excellent tumor control while significantly lowering normal-lung toxicity in a variety of preclinical models and early human experience. Its protective effects, caused by processes such as transitory oxygen deprivation, altered redox chemistry, mitochondrial preservation, immunological modulation, and vascular stabilization, indicate a genuine widening of the therapeutic window beyond what standard-dose-rate radiation can achieve. Parallel advancements in proton PBS delivery, Bragg peak engineering, TB techniques, adaptive planning, and advanced MC optimization have dramatically enhanced the capability to deliver UHDR with millisecond precision in the dynamic thoracic environment.

Despite these advancements, various obstacles preclude immediate practical application. Standardized dose rate measurements, dependable real-time dosimetry, UHDR-capable beamline availability, and robust motion-management solutions remain unmet needs. Biological variability and insufficient long-term toxicity data highlight the importance of adequately designed, multicenter clinical trials. Finally, FLASH-RT's promise of safer, more successful lung cancer treatment will depend on ongoing interdisciplinary collaboration to define ideal settings, validate mechanistic pathways, and develop evidence-based guidelines to enable its implementation in routine clinical practice.
